# Unsuccessful mitosis in multicellular tumour spheroids

**DOI:** 10.18632/oncotarget.15673

**Published:** 2017-02-24

**Authors:** Annie Molla, Morgane Couvet, Jean-Luc Coll

**Affiliations:** ^1^ Institute for Advance Biosciences, Centre de Recherche UGA, INSERM U1209, CNRS UMR 5309, 38700 La Tronche, France

**Keywords:** spheroid, mitosis, cytokinesis, mitotic drug, tetraploid cells

## Abstract

Multicellular spheroids are very attractive models in oncology because they mimic the 3D organization of the tumour cells with their microenvironment. We show here using 3 different cell types (mammary TSA/pc, embryonic kidney Hek293 and cervical cancer HeLa), that when the cells are growing as spheroids the frequency of binucleated cells is augmented as occurs in some human tumours.

We therefore describe mitosis in multicellular spheroids by following mitotic markers and by time-lapse experiments. Chromosomes alignment appears to be correct on the metaphasic plate and the passenger complex is well localized on centromere. Moreover aurora kinases are fully active and histone H3 is phosphorylated on Ser 10. Consequently, the mitotic spindle checkpoint is satisfied and, anaphase proceeds as illustrated by the transfer of survivin on the spindle and by the segregation of the two lots of chromosomes. However, the segregation plane is not well defined and oscillations of the dividing cells are observed. Finally, cytokinesis fails and the absence of separation of the two daughter cells gives rise to binucleated cells.

Division orientation is specified during interphase and persists throughout mitosis. Our data indicate that the cancer cells, in multicellular spheroids, lose their ability to regulate their orientation, a feature commonly encountered in tumours.

Moreover, multicellular spheroid expansion is still sensitive to mitotic drugs as pactlitaxel and aurora kinase inhibitors. The spheroids thus represent a highly relevant model for studying drug efficiency in tumours.

## INTRODUCTION

For several decades, two-dimensional- (2D)-cell cultures were used for the screening of anti-cancer drugs, however they do not fully recapitulate the solid tumour architecture and its biology. Cells grown as monolayer, on 2D surfaces, present few cell-cell and cell–matrix interactions with a stretched and abnormal cytoskeleton rearrangement that lead to aberrant gene and protein expression. Additionally, the cells are uniformly oxygenated with a full access to nutriments. These changes result in accelerated doubling times. Three-dimensional (3D)-cultures emerged that realistically recapitulated tumour cell behaviour and have become, therefore, increasingly popular, in recent years. The architecture of 3D-cultures reflects the microenvironment of solid tumour with appropriated cell-cell interactions as well as gradients of nutrient and oxygen [1]. 3D-multicellular spheroids include proliferating, quiescent and hypoxic regions and, display very different sensibilities towards anti-cancer drugs and a more realistic anti-cancer drug response model compared to 2D cultures [2]. 3D systems also offer the opportunity to co-culture different types of cells, such as tumour cells plus as well as stromal fibroblast and immune cells. Complex spheroids also mimic the microenvironment of the tumour and, compared to mono-cellular spheroids, different biochemical pathways are activated. As an example, the presence of cancer-activated fibroblasts within melanoma spheroids was shown to prevent ERK/MAP signalling and to re-activate FAK proliferating pathway [3].

Several systems of 3D-cell culture have been developed [4]. In the simplest one, the free floating spheroid, tumour cells are allowed to aggregate on a non-adherent support [5]. This system was then modified in order to introduce an extracellular matrix (ECM), which is a key regulator of normal homeostasis and tissue phenotype. Laminin-rich extracellular matrix and basement-membrane extracts (matrigel™) were used to grow the cells within or on-top of these matrix [6, 7]. Alternatively, tumour cells have been encapsulated in alginate hydrogels [8]. Alginate is a natural polymer that provides an inert 3D-environment. It can be mixed with ECM and allow to study the aggressiveness of tumours as a function of the matrix stiffness [9]. Organoid models are also developed from cancer tissues: stem cells are isolated and embedded in ECM. Such reconstituted organoids have similar heterogeneities to the original tumours [10].

These 3D systems are valuable tools for anti-cancer drug testing [11]. Comparison of gene and protein expression reveals that metabolic, cell stress-response, structural, signal transduction, and cellular transport proteins are expressed at different levels in spheroids as compared to 2D-cultured cells [12] and [13].

In growing spheroids, the proliferating cells are preferentially located in the outermost layers [14]. Previous reports described the cell cycle parameters but none has focused on the progression of mitosis [14]. Mitosis allows the segregation of the two lots of chromosomes and finally the separation of the two daughter cells. Mitosis is a highly regulated process controlled by many regulatory complexes such as the crucial passenger complex (aurora-B kinase, survivin, …) [15]. The cells must satisfy two successive checkpoints: the spindle checkpoint that prevents the cells from entering in anaphase and the noCut checkpoint that controls the division of the cytoplasm (cytokinesis) [16, 17].

In the present study, we report the presence of bi-nucleated cells in multicellular spheroids and we describe mitosis progression. The cell cycle analysis of 7 days old-spheroids indicated a decreased S-phase and a stable 4N-population that represents mitosis-arrested and tetraploid cells.

We set-up time-lapse experiments on live fluorescent spheroids to follow mitosis. Direct live imaging has been poorly explored on spheroids. Our study demonstrates that the mitotic spindle checkpoint is satisfied since chromosomes are aligned on the metaphasic plate, histone H3 is phosphorylated on Ser10 and the passenger complex is transferred on the spindle. Conversely, cytokinesis failed as a consequence of the mis-orientation of the dividing axis, giving rise to binucleated cells.

Despite unsuccessful mitosis, multicellular spheroids are a suitable model for testing mitotic drugs because their expansion is sensitive to pactlitaxel and aurora kinase inhibitors. Spheroids, thus, represent a highly relevant model for studying drug efficiency.

## RESULTS

### Presence of binucleated cells in spheroids

**Figure 1:**
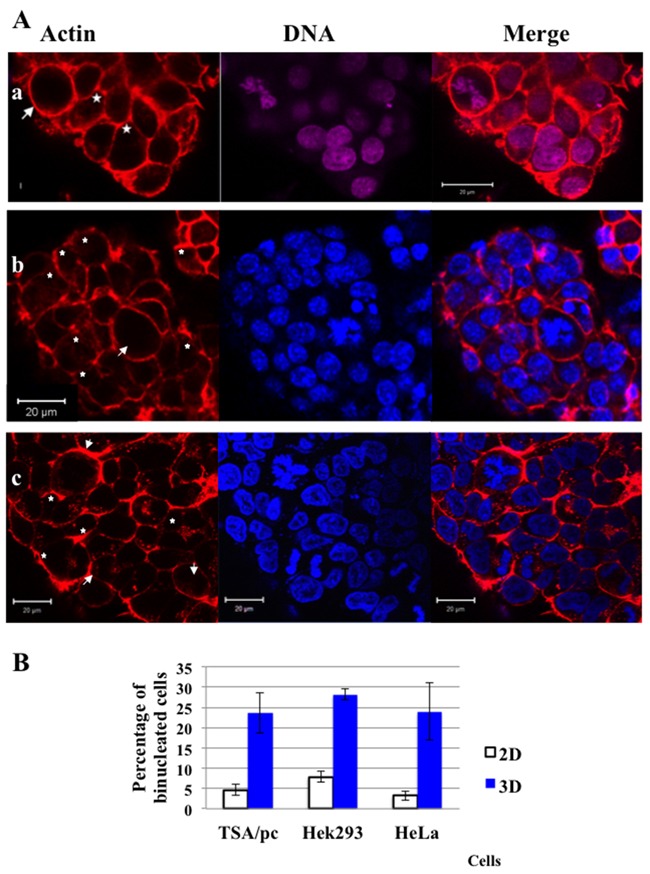
Cytokinesis failure and presence of binucleated cells in spheroids **(A)** In **(a)** TSA/pc spheroids were grown for 7 days in U-low binding plates; In **(b)** TSA/pc spheroids prepared by the pending drop technic and recovered for staining on day 7. In **(c)** Hek293 spheroids were grown, for 7 days, in U-low binding plates. The outlines of cells are highlighted by labelling actin with rhodamin-phalloidin (a, b and c). DNA was labelled by either NucRed in (a) or hoechst 33342 in (b) and (c). Mitotic cells are indicated by an arrow and binucleated cells by stars. The bar represents 20 μm. **(B)** Estimation of the percentage of binucleated cells in three cell lines. 3 200 and 1 650 TSA/pc, 750 and 640 Hek293 and finally, 889 and 1 544 HeLa cells were scored on 2D-cultures and in D7-spheroids, respectively. Results obtained with 2D-cell cultures are represented by white histograms whereas 3D-spheroids data are in blue. The differences between 2D and 3D were found significant (p < 0.05 for Hek293 and < 0.005 for others as determined by student test).

Murine, p53WT TSA/pc cells derived from a mammary gland tumour were used for 3D-MCTS production either in U-low binding 96-wells or by the pending drop method. In both cases we identified a significant proportion of bi-nucleated cells within the spheroids (Figure 1A (a) and (b) respectively). The same observations were made with Hek293 (Figure 1A (c)) and HeLa spheroids (not shown). The proportion of bi-nucleated cells at the spheroid surface was 23.7, 28.2 and 24.0 % respectively for TSA/pc, Hek293 and HeLa cells (Figure 1B). The proportions of bi-nucleated cells increased by around 20 % when cells were grown as spheroids compared to 2D-plastic cultures (Figure 1B).

### Detection of mitotic markers

**Figure 2:**
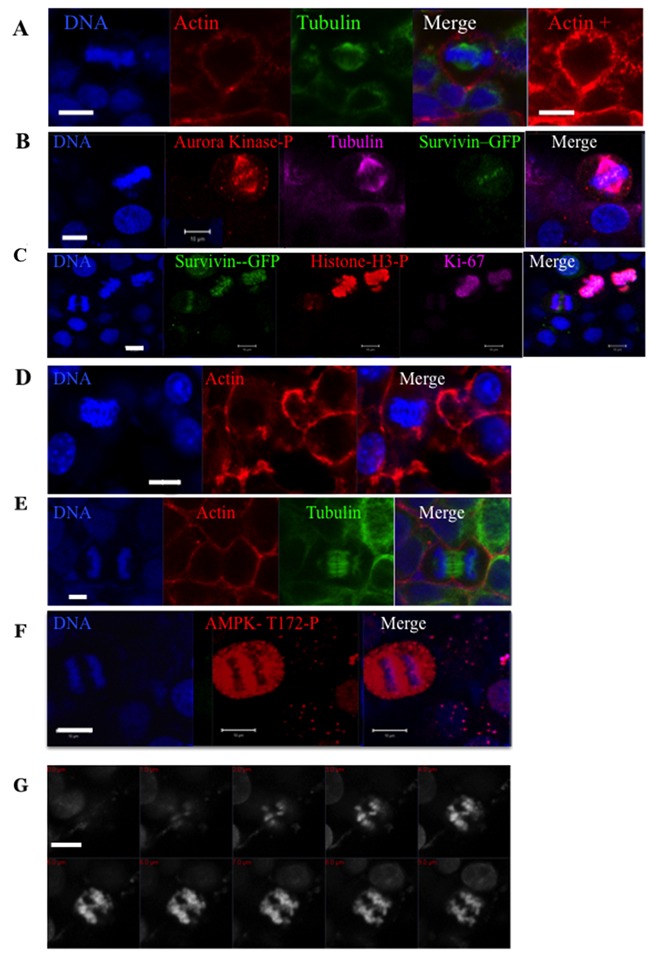
Immunofluorescence experiments on whole HeLa spheroids Cells in pro-metaphase are shown in **(A-C)** and labelled with anti-tubulin (A and B) and phalloidin in (A). In (B) survivin is detected by its GFP tag and the activities of aurora kinases A-B-C by a specific phospho-antibody (in magenta). In **(C)**, several mitotic cells detected by survivin-GFP are labelled by [KI67] (in magenta) and Ser10-phospho-histone H3 (in red) antibodies. Early and late anaphases are imaged in **(C and D)** and **(E-F)** respectively. The localisation of actin (in red) and tubulin (in green) are shown in (D) or (E). Phospho-AMPK (T172) is detected in (F). In **(G)**, a collection of images corresponding the z-imaging of the DNA of an anaphase is shown. In all panels, DNA is labelled by hoechst 3342 and the bar represents 10 μm.

Since the presence of bi-nucleated cells is a consequence of unsuccessful mitosis, we studied the localization of important mitotic proteins and markers (Figure 2). We performed immunofluorescence experiments on HeLa cells either WT or stably expressing survivin-GFP (Figure 2). Survivin is a member of the chromosomal passenger complex that controls the spindle checkpoint, first mitotic checkpoint [18]. Other members of this complex are InCenp, borealin and aurora-B kinase. In spheroids, mitotic cells were imaged. The chromosomes are aligned on the metaphasic plate (Figure 1A (a)) and the mitotic spindle appears normal (Figure 2A-2C). As expected, survivin-GFP was located on the centromeres and the aurora B kinase was fully active since it was found to be phosphorylated in 100% of the mitotic cells (46 cells scored) (Figure 2B). The phosphorylation of Histone H3 on Ser10 was also detected in 100% of metaphases (93 counted). This signal is absolutely required before the anaphase can proceed [19]. As expected also, Ki67 was decorating the metaphasic chromosomes (Figure 2C). According to these data, we can conclude that the spindle checkpoint is satisfied and that the anaphase can proceed. Anaphases were detected in the spheroids (Figure 1A (c) and Figure 2C-G) and both actin and tubulin appeared properly localized. Survivin-GFP was transferred on the spindle (Figure 2C) and the phosphorylation of AMPK-Thr172 was detected on late anaphase as expected (Figure 2F) [20]. In comparison to mitosis occurring in 2D cultures, we noted that some metaphases were not perfectly round (Figure 2A) and anaphases appeared in contact with neighbour cells (Figure 2D and 2E). In anaphase, we often observed lagging chromosome and chromosomes appeared twisted (Figure 2G).

### Time-lapse experiments

**Figure 3:**
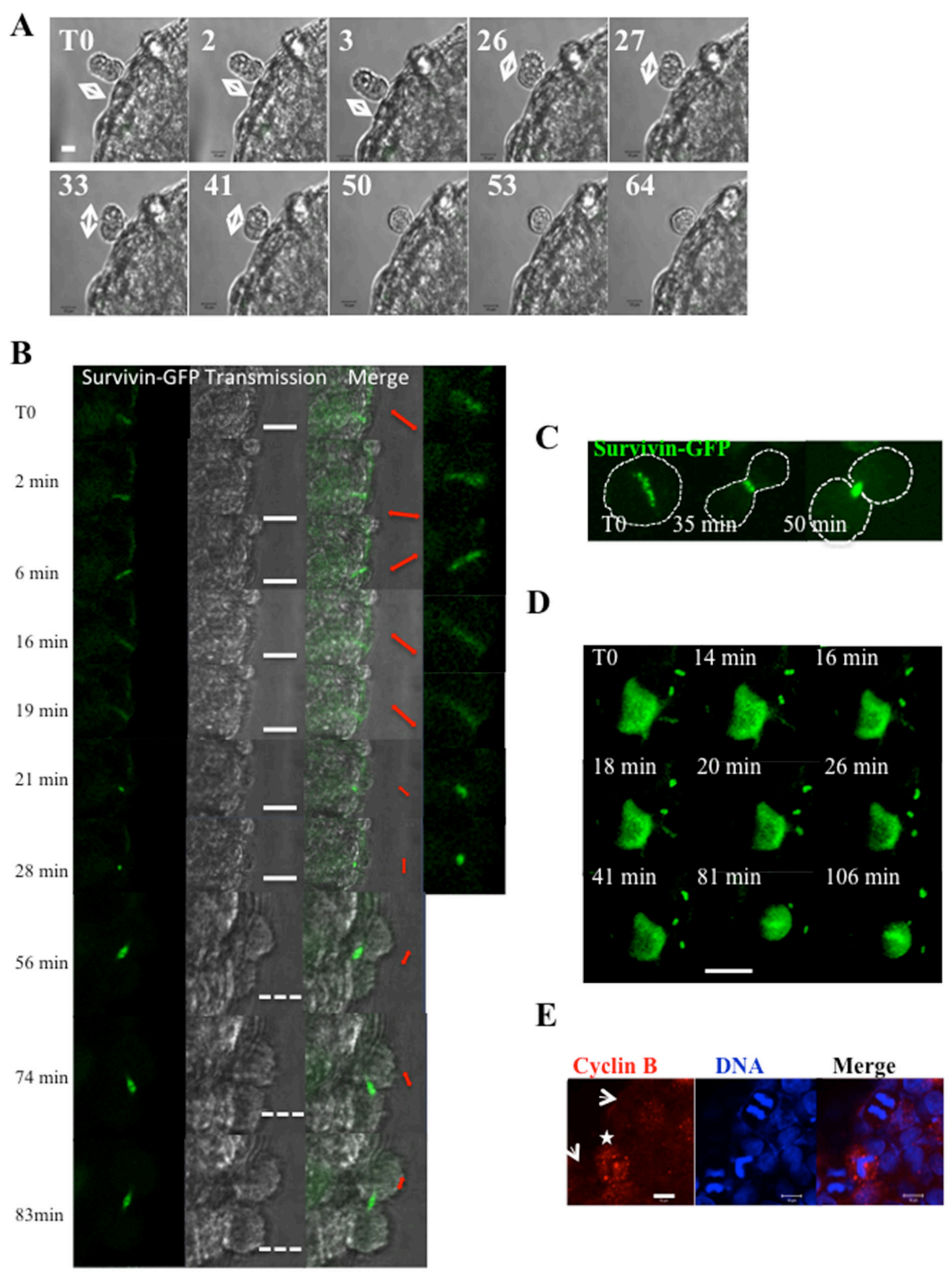
Time-lapse experiments on whole HeLa spheroids **(A)** An anaphase is detected by its peculiar shape at the border of a Hek293 spheroid and the spheroid was continuously imaged. Elapsed times in hours are indicated on the top left of images and the direction of the long axis is denoted by arrows. The bar represents 10 μm. **(B)** Cells stably expressed survivin-GFP. An area including a metaphase is continuously imaged. Elapsed times are indicated. For each time point, the fluorescent image and the brightfield are shown. A merge and a 2.25-zoom are added on the right; the direction of the fluorescent signal is indicated in red (metaphasic plate from T0 to 28 min and then midbody). The white bar represents 20 μm and the discontinuous one 40 μm. **(C)** Time-lapse of survivin-GFP in cells grown in 2D. The metaphasic cell enters in anaphase 35 min later and is in telophase at t= 50 min. Based on the fluorescent background a discontinuous line was drawn to figure out the shape of the cell. **(D)** Same experiment than in B. This larger field included one metaphasic cell and several telophases. The white bar represents 20 μm. Elapsed times are indicated on each photo, T0 been the starting point. **(E)** Detection of cyclin B on HeLa spheroids. Cyclin B appears in red and DNA detected by hoechst3342 in blue. The arrows point anaphases and a star indicates a metaphase. The bar represented 10 μm.

At the spheroid periphery, a cell in anaphase identified by its shape was continuously imaged (Figure 3A). This time-lapse revealed movements of the two daughter cells (Figure 3A, T0 to 41 min) and the rotation of the division axis. Finally, a unique round cell was obtained due to the absence of segregation (Figure 3A, 50 min to 64 min). This defect of separation of the two daughter cells may lead to the presence of bi-nucleated cells in spheroids.

In order to better describe the mitosis, we performed time-lapse experiments on HeLa cells expressing survivin-GFP. Survivin is a passenger protein localized on the centromeres as recalled in Figure 3C (T0). At anaphase, survivin is transferred on the central spindle (Figure 3C, 35 min) and it lies on microtubules till the final segregation (Figure 3C, 35 min). Survivin-GFP was detected within the spheroids and time-lapse experiments were realized as follows (Figure 3B). In each experiment, an area encompassing one metaphase is imaged continuously (Figure 3B). In 2D cultures, the spindle predetermined the direction and the orientation of the future segregation (Figure 3C). Within growing spheroids, the fluorescent signal was found punctuated from T0 to 6 min. Then, survivin was transferred on the spindle and was detected as small rods (Figure 3B, t= 16 min and 19 min). Finally, a clear midbody appeared (Figure 3B, t= 56 min to 83 min). However the metaphasic plate (schematically represented by the red arrows from T0 to 28 min) was found to rotate. This is an unusual feature. Changes of orientation of the midbody were also not expected (Figure 3B, t 56 min to 83 min). This rotation of the metaphasic plate (Figure 3D T0 to 18 min) and of the midbody (Figure 3D) was observed in all cases (see field in Figure 3D). The changes of orientation of the midbody are consistent with the movements of the two daughter cells observed in Figure 3A (T0 to 41 min).

The transfer of survivin from chromatin to microtubules indicates that the spindle checkpoint is satisfied when the cells are growing as spheroids (Figure 3B). As expected, we therefore observed a decrease of cyclin B in anaphases (Figure 3E), corresponding to its degradation.

**Figure 4:**
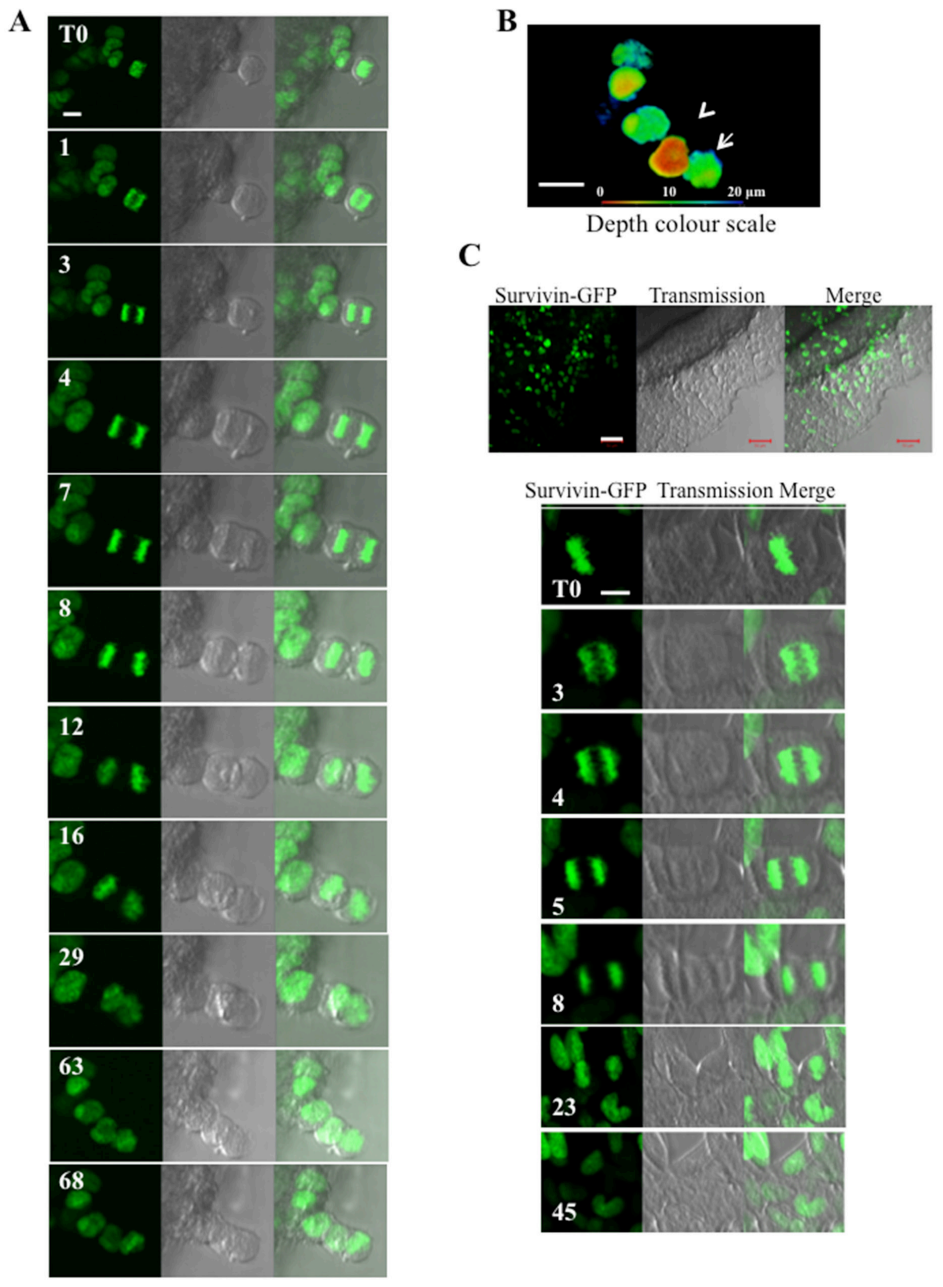
Time-lapse experiments on whole Hek293 spheroids **(A)** Hek293 cells expressing histone H2A-GFP are continuously imaged. An early anaphase is selected at T0. The fluorescent signal of histone H2A-GFP, the brightfield and the merge are shown at representative times. **(B)** A z-stack imaging of the field shown in (A) was performed at T68. The maximum intensity projection of the whole fluorescent signal is represented with a depth colour code. Arrows indicates the two nuclei derived from the anaphase imaged in A (one brown, one green). **(C)** Same time-lapse as in A performed on whole spheroids kept for one day in Labteck wells. The border cells start to spread on the glass as seen in (a) and the metaphase cells escaping from the spheroid mass undergo normal in mitosis.

To better define these unexpected features, we performed a time-lapse on cells expressing histone H2A-GFP (Figure 4A). We could not work with a clonal population that was too bright. For accurate results, we worked with a population in which about 30% of the cells were fluorescent. Early anaphases were selected at T0 and continuously imaged. As shown in Figure 4A, the two lots of chromosomes started to separate at 1 min, but the orientation of the anaphase was different 2 minutes later (3 min). At 8 min, the chromosomes were already condensed and the cells started to segregate, however, segregation was finally not successful. The anaphase (T0) gave rise to a unique cell with two nuclei (Figure 4A) that appeared in two different z-plans (projection in Figure 4B). The absence of segregation was a consequence of mitosis progression in the spheroid or at its surface. If the same spheroids were allowed to spread overnight on glass, normal separations occurred (Figure 4C). This suggested that, in the spheroid, cytokinesis failure is either due to the contact with the neighbouring cells or to unstretched cytoskeleton in interphase.

**Figure 5:**
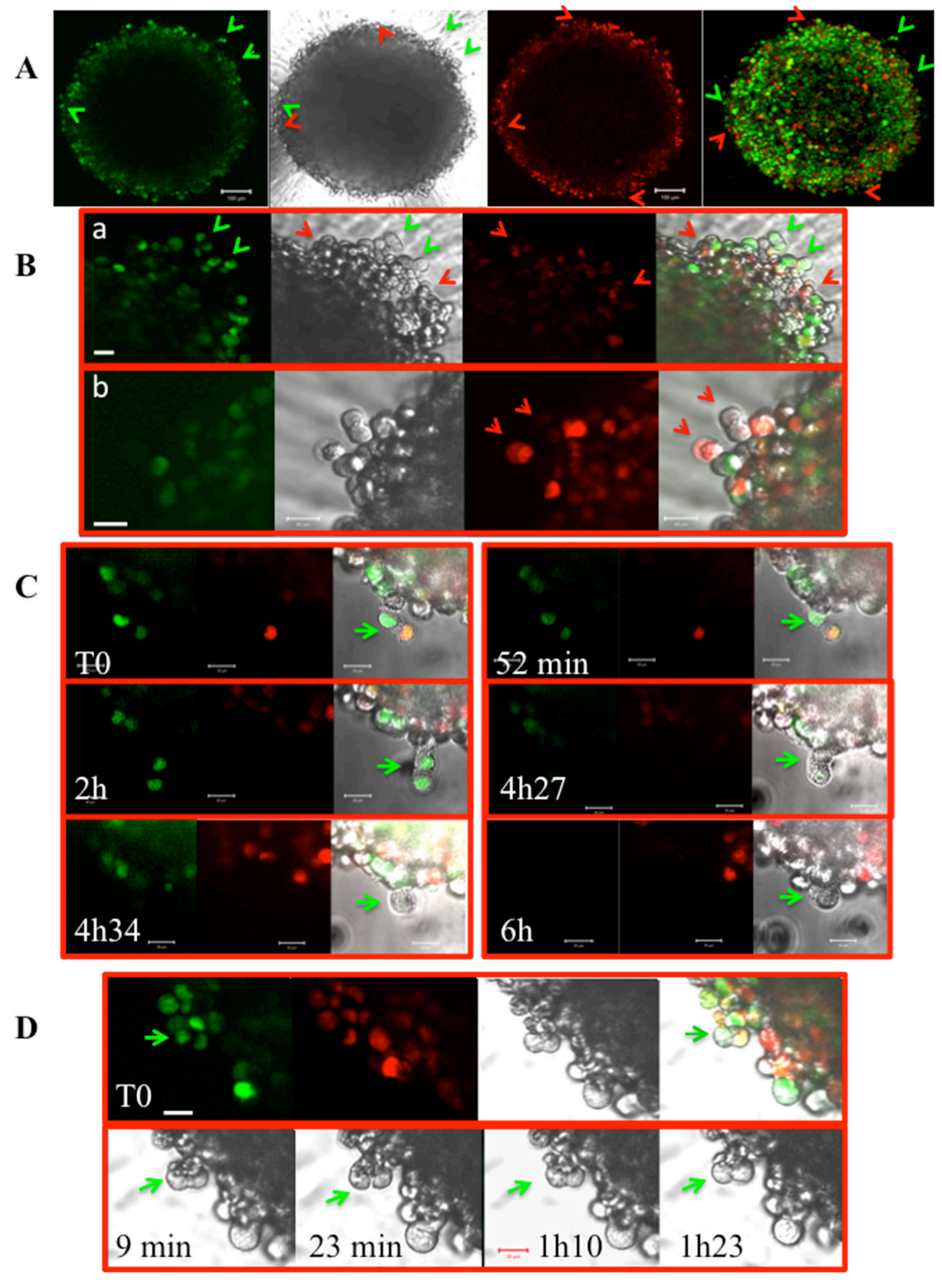
Experiments on Fucci-HeLa cells **(A)** A Fucci-HeLa spheroid grown for 7 days is imaged directly in the well. Green cells expressing geminin-GFP are mostly in G2/M or in late S when red cells expressing cdt1-RFP are in G1 to S. Binucleated green and red cells are indicated by the corresponding coloured arrows. A brightfield and a merge (green and red) are shown. The bar corresponded to 100 μm. **(B)** Same as in A under higher magnitude. The bar corresponded to 20 μm. In **(a)**, note the presence of green mitoses and red binucleated cells and in **(b)** of a mostly black mitosis and a binucleated cell. They are indicated by arrows. **(C)** Time-lapse on a Fucci-HeLa spheroid grown for 7 days. An anaphase is selected by its peculiar shape at the border of the spheroid and continuously imaged. Elapsed times are indicated. This anaphase looses it green colour at t= 4h27 and finally gives rise to a unique uncoloured cell (4h 34 and 6h). The bar corresponded to 20 μm. **(D)** Time-lapse on a NMuMG-Fucci spheroid grown for 7 days. An anaphase is selected by its peculiar shape at the border of the spheroid and continuously imaged for 1h20. A green arrow indicates its position. Elapsed times are indicated. The bar corresponded to 20 μm.

To ascertain that cells escape from mitosis, experiments were performed with Fucci-HeLa cells expressing the two sensors: geminin-GFP and cdt1-RFP [21]. Geminin is mostly expressed in late S-phase and G2/M whereas Cdt1 peaks in G1 and declines after the initiation of S-phase. These markers allow ascertaining that the cells initially identified by their shape as anaphase (Figure 3A) are really mitotic cells with two lots of chromosomes (Figure 5A and 5B). The presence of red bi-nucleated cells (Figure 5A and 5B) fits with the time-lapse observations (Figure 3 and 4). G2/M-green and G1-red binucleated cells were detected at the surface of the spheroids (Figure 5A and B). The presence of red binucleated cells confirmed that the cells escaped from mitosis without segregation. In part C, a time-lapse was performed on Fucci-HeLa cells. At T0, an anaphase was selected (pointed with a green arrow) and imaged for around 6 hours. We observed a movement of the future daughter cells (compare 52 min with 2h) from the right to the left. Finally, this mitotic cell gives rise to a large non-coloured cell in early G1 phase (Figure 5C, 4h34 and 6h). When a similar experiment was conducted on Fucci-NMu-Mg cells, we did not observe any movement of the future daughter cells pointed with a green arrow, for a period of 1h23 (Figure 5D). Meanwhile, the increase of polyploid cells was non-statistically significant in these normal mammary gland cells (9.5 % ± 2.8 in 2D cultures compared to 10.1 ± 4.5 in spheroids).

### Role of fibronectin

**Figure 6:**
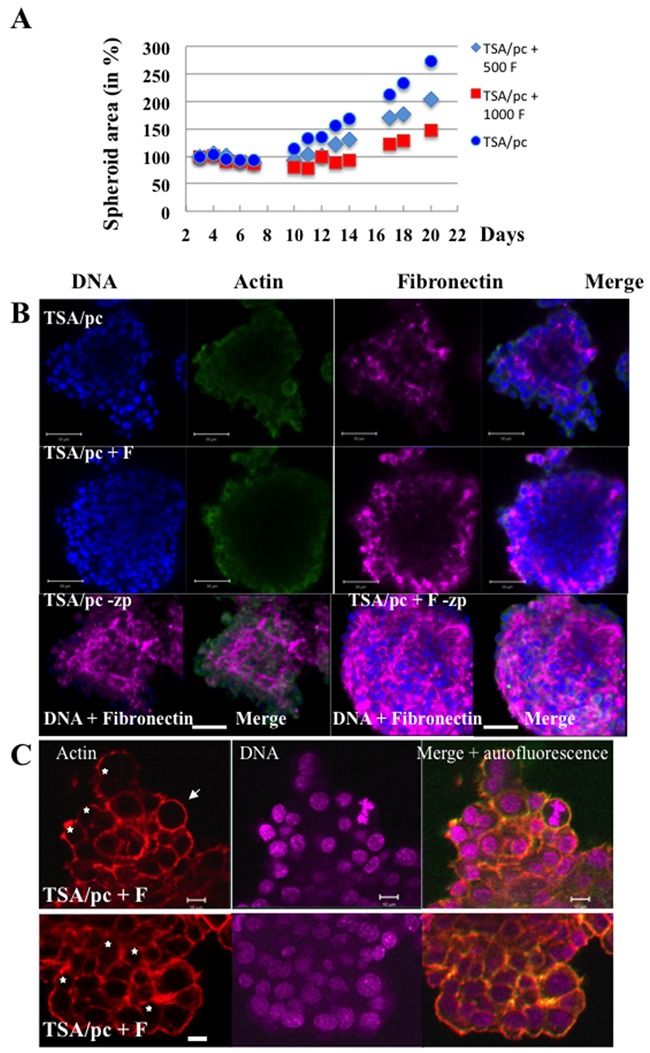
Bi-nucleated cells in mixed spheroids **(A)** mixed spheroids were established with TSA/pc cells (1000) and 3T3-fibroblasts (500 F or 1000F). The expansion of the spheroids was followed over time. **(B)** immunofluorescences were realized on whole spheroids and fibronectin was detected (shown in magenta). DNA and actin fluorescent signals were imaged on spheroid optical sections as fibronectin was also detected (shown in magenta). A 3D projection of the whole fluorescence is shown and indicated as –zp. The bars represent 50 μm. **(C)** Binucleated cells are detected in mixed spheroids and indicated by stars. The white arrow highlights a metaphase. The bars represent 10 μm.

One possible explanation for the mis-orientation of the cytokinesis axis could be a lack of tension between the daughter cells. We looked for the organization of fibronectin, an extracellular matrix protein. TSA/pc spheroids were expanded continuously (Figure 6A). When mixed spheroids were prepared with tumour cells and fibroblasts, they grew slowly and they were more compact than spheroids made of tumour cells only (Figure 6A). The detection of fibronectin indicated differences in its organisation (Figure 6B). Surprisingly, TSA/pc-spheroids did not assemble fibronectin at their border (Figure 6B, TSA/pc-zp). The opposite could be expected since only exterior cells were in contact with serum. In mixed spheroids, the signal of fibronectin significantly increased and it was detected on the whole spheroids (Figure 6B, TSA/pc +F-zp). In mixed spheroids, cells were embedded in fibronectin and the structure was compact but we still detected binucleated cells (Figure 6C).

### Cell cycle analysis

**Figure 7:**
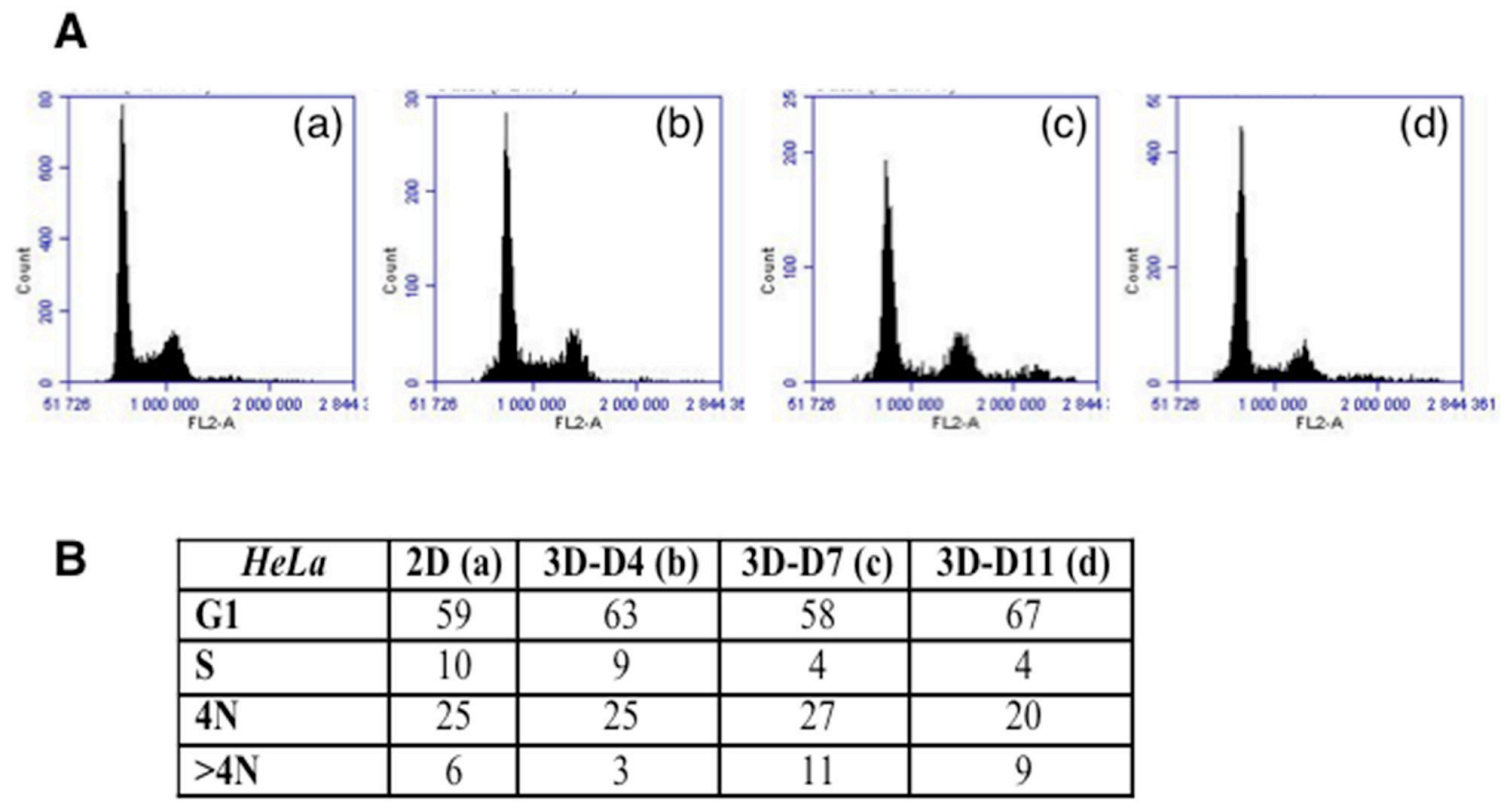
Cell cycle analysis on TSA/pc cells **(A)** Histograms of the distribution of the cells grown as adherent cells **(in a)** or as spheroids **(in b-d)**. The spheroids were recovered on day 4, 7 and 11. **(B)** Estimation of the percentage of cells in the different phases.

The analysis of the cycle revealed a decrease of S-phase; this phase represented 9 %, on day 4 and 4 % on day 7, for cells grown as spheroid (Figure 7). This reflects the proliferation rate within the spheroids. Meanwhile the 4N fraction was still high (27 % on day 7 and 20 % on day 11) but it was accounted for by interphasic bi-nucleated and G2/M cells. Moreover, the mitosis was found to be slowed down as illustrated by time-lapses (Figure 4A). The sup >4N fraction increased progressively to reach the initial level of the cell line in 2D culture (Figure 7). These data suggested that binucleated cells mostly did not enter in a new cycle.

### Role of p53

**Figure 8:**
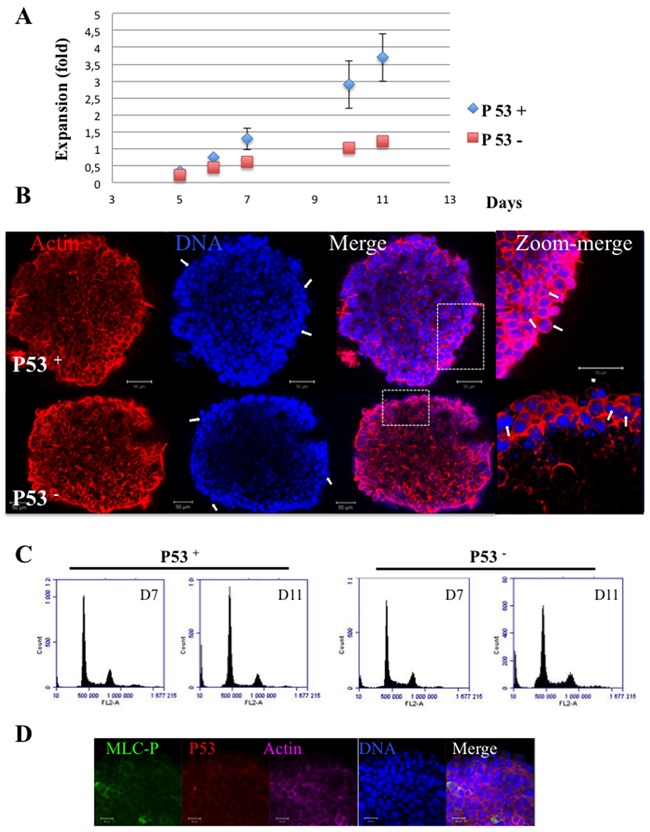
Hct116 (P53 + and P53-) Spheroids **(A)** Spheroids were established and their growth was followed for 11 days. Expansion at Dx equals (area Dx – area D0/area D0). In 2D-cultures Hct116-P53-cells grew 20 % less rapidly than the P53+ cells (data not shown). **(B)** Actin and DNA are labelled by phalloidin and hoechst 33342 respectively; Some bi-nucleated cells on the surface are indicated by arrows. The provided zooms corresponded to the area indicated by rectangles. The bars represent 50 μm. **(C)** Cell cycle of Hct116-(P53+ and P53 -) cells grown as spheroids for 7 and 11 days. Percentages of polyploid cells of 6.8 ± 1.4 and 5.1 ± O.9 were found for Hct116-(P53+ and P53 -) cells respectively, in 2D cultures. **(D)** Immunofluorescence of TSA/pc spheroids: detection of MRLC-P (in green) and P53 (in red) in day 7 spheroids.

In adherent cells growing in 2D, P53 was described to be stabilized in bi-nucleated cells and to prevent the proliferation of tetraploid cells [22]. Since the increase of bi-nucleated cells was observed in P53 positive cells (Figure 1), we describe the situation in P53 negative cells. We prepared spheroids from genetically modified hct116 cells expressing P53 (P53+) or not (P53-). P53+ spheroids grew faster than P53- ones similarly to results obtained in 2D cultures (Figure 8A). Immunofluorescence experiments revealed, on both types of spheroids, the presence of binucleated cells and the absence of larger polynucleated cells (> 4 N) (Figure 8B). The comparison of cell cycle of both spheroids did not reveal major differences and the > 4 N fractions were very similar in both, even at day 11 (Figure 8C). In addition, we observed no stabilisation of p53 in hct116 cells (data not shown) and in TSA/pc cells (Figure 8D). These results and the lower expansion of P53- spheroids compared to P53+ ones as well as the similar > 4N phases suggest that P53 is probably not involved in the proliferation arrest of binucleated cells. This arrest could only reflect the low proliferation rate within the spheroids as illustrated by the decrease in percentage of cells in S-phase (Figure 7 and 8C).

### Test of antiproliferating drugs targeting mitosis

Spheroids are proposed as models for testing anti-cancer drugs since they better mimic tumours than adherent cells. Taking into account cytokinesis failure, we questioned whether the system was still suitable for testing mitotic drugs. We selected three anti-mitotic compounds: paclitaxel that prevents tubulin depolymerisation [23], AZD1172 [24] and C5M [25] two aurora B-kinase inhibitors (Table 1). In addition to aurora B inhibition, C5M targets the noCut checkpoint by preventing AMPK-172 phosphorylation [25]. The three drugs inhibited the expansion of TSA/pc spheroids with IC50 similar to those measured in 2D assays except for C5M. The IC50 of C5M was 2.5 higher in 3D. Their efficiencies decreased on ‘older’ spheroids, around 6 days post establishment, in which less cells were in mitosis. The IC50 increased from 20 nM to 35 nM for paclitaxel and from 25 to 150 nM for AZD1172. Although cytokinesis failed in spheroids, the model was thus found relevant for screening different anti-proliferating drugs targeting mitosis.

**Table 1:** Efficiency of mitotic drug on TSA/pc spheroids compared to 2D

Culture		2D	3D	
Drug added at		24 h	96 h	156 h
Treatment duration		96 h	5 days	7 days
**Drugs**	Paclitaxel	12 ± 2 nM	20 ± 5 nM	35 ± 5 nM
	AZD1172	32 ± 5 nM	25 ± 6 nM	150 ± 25 nM
	C5M	187 ± 22 nM	438 ± 12 nM	ND

± stands for standard deviation.

As a conclusion, we observed cytokinesis failure, in different cell-spheroids, as a consequence of the destabilization and mis-orientation of the segregation axis.

## DISCUSSION

Three-dimensional (3D) culture systems are valuable tools for anti-cancer drug testing since cells acquire morphological and cellular characteristics relevant to *in vivo* tumours [2]. Among the different available systems we chose the free-floating spheroid for its easy handling and the possibilities of microscopy. We imaged daily the spheroids grown in U-well plates, under live conditions, and we followed each spheroid individually. These spheroids assemble their own matrix and TSA/pc spheroids grow exponentially for at least three weeks. As also reported by other authors [26, 27], the analysis of the cell cycle revealed a large decrease of the S-phase within the spheroid that is consistent with a doubling time of the whole population in around 7 days. This decrease of S-phase did not fit with a quite constant presence of G2/M cells. The presence of a large proportion of bi-nucleated cells could account for this 4N-fraction. This tetraploid population was observed in spheroids prepared with different cell lines and even in compact spheroids generated by addition of fibroblasts [28]. Polyploidisation of cells grown in suspension was only reported, in 1982, for chinese hamster V-79 cells which spontaneously formed spheroids [29].

We decided to describe the progression of mitotic cells at the periphery of the spheroid. Many different imaging, like classical and biphotonic fluorescent microscopy [30] and complex imaging such as light sheet (LS), were performed on spheroids [31]. However, to our knowledge, direct time-lapse experiments without a reconstitution step have not yet been reported in spheroids. We adapted to 3D-cultures the fluorescent time-lapse experiments widely used in 2D-cultures by acquiring images on a confocal microscope using a Plan-Apochromat 20X/0.75 objective. As in 2D-cultures, this technique allowed to describe step-by-step the progression of mitosis. We found that the passenger complex was well localized on the centromere and was fully active. The spindle checkpoint was thus satisfied and anaphase proceeded as accounted for by the transfer of survivin-GFP on the mitotic spindle. Meanwhile the two lots of chromosomes were separated. In 2D-cultures, the anaphase cells had the same orientation as in the former metaphase and the cytokinesis was thus already oriented. At the periphery of the spheroids where most division occurred, we observed continuous movements of the mid-body. The absence of stabilization of the furrow division and the presence of chromatin in the segregation plane prevented the separation of the two-daughter cells. After a long arrest, cells escaped from mitosis and gave rise to a G1-binucleated cell. We supposed that, because of an active proteolysis and in the absence of transcription, some proteins are in too low concentration for maintaining mitosis. Cytokinesis failure was thus responsible for the increase of binucleated cells. Conversely to what was reported for 2D-culture, cytokinesis failure did not induce the stabilisation of p53 and presumably did not activate the hyppo tumour suppressor pathway [22, 32]. In spite of these unsuccessful mitoses, free floating spheroids are a valuable system for evaluating mitotic drugs even when these drugs target late events.

Cytokinesis failure could be the consequence of the destabilization of the axis of division. The axis of division is influenced by the interaction of spindle microtubules with cortical actin, by forces generated at the cellular cortex and by the shape of the cell [33, 34, 35]. In fact, the distribution of retraction fibres during mitosis constitutes a memory of the adhesion pattern in interphase and controls the orientation of the spindle [36]. In the spheroid, we noted that few mitotic cells were not round and had unusual contacts with the surrounding cells. In many disease processes including cancer, cells may lose their ability to regulate spindle orientation [36, 37] giving rise to aberrant structures [38]. Cancer multicellular spheroids recapitulate such a feature that was not found in one example of normal mammary cells. More investigations are needed for establishing a link between the transformation and the polyploidisation of cells grown as spheroid.

Accumulating evidence points the significant contribution of tetraploid intermediate in the composition of cancer genomes: about 20% of all solid tumours exhibit tetraploid karyotypes [39]. Moreover bioinformatics analyses on 4 000 human cancers revealed that about 37 % of all tumours, even with diploid karyotypes, have undergone at least one whole genome duplication event during their evolution [40]. Spheroids represent an attractive model for understanding such events. Especially as tetraploid cells are genetically unstable and can promote tumorigenesis [32].

## MATERIALS AND METHODS

### Cells

HeLa and Hek293 cell lines obtained from ATCC were cultured under the suggested conditions. HeLa cells stably expressing survivin-GFP and Hek-293 expressing histone H2A-GFP were already described [41] [42]. TS/A-pc cells were cultured in RPMI 1640 media supplemented with 1% glutamine. Genetically modified HCT116 (P53+ and P53-) cells were a gift from Dr B. Vogelstein. Fucci-HeLa and Fucci-NMuMG cells expressed both cdt1-RFP and geminin-GFP [21].

### Compounds

Paclitaxel and AZD-1172 were supplied by Sigma-Aldrich. C5M was described in Le et al [25].

### Multicellular tumour spheroid models

Spheroids were generated by plating either TS/A-pc or HeLa or Hek293 or Hct116 cells, at 1000, 800, 500 and 1000 cells/well respectively, into ultra low adherence −96-well plates (Corning, Tewksbury, USA). These plates stimulate spontaneous formation of a single spheroid of cells, within 24 hours of incubation at 37°C, 5% CO_2_. Spheroids grew in complete medium as in 2D-cultures in the final volume of 200 μL.

Mixed spheroids containing both TSA/pc tumour cells and NIH-3T3 fibroblasts were essentially prepared as already described in [25] except that both cells are mixed at the starting point.

Alternatively spheroids were prepared by the hanging drop technic. Drops of 40 μL (around 10 000 cells /mL, in culture medium with 10% FCS) were pipetted on the lid of a petri dish containing PBS. The culture was maintained for 5 to 7 days.

Spheroids were imaged in live conditions either directly in the 96-well plate or upon their transfer into coverglass Lab-Tek wells.

For measuring their expansion, spheroids were imaged with a Zeiss510-confocal microscope, each day, with a 10-X objective and then the area of the larger section was determined for each spheroid by the Image J software. Most experiments were done on 5 spheroids alternatively on 3. Finally, at the end-point, the number of live cells was determined by the celltiter Glo-3D kit under conditions suggested by the manufacturer (Promega).

### Cell cycle analysis

For determination of cell cycle profiles, adherent cells were recovered and fixed by ice-cold 70% ethanol, for 1 hour, and then, incubated with propidium iodide solution (50 μg/ml) in the presence of 0.2 mg/ml RNase A for 15 minutes, at 37°C. DNA content was measured using the BD Accuri C6 flow cytometer (BD Biosciences, US) and CFlow Plus software.

Determination of the cell cycle of cells grown as spheroids proceeded as follows: Five spheroids were recovered by pipetting in an eppendorf tube, washed by PBS then, transferred in a well of a 24-plate containing trysin/EDTA, incubated at 37°C with vigorously shaking from time to time. The dislocation of the spheroids is followed under the microscope and the action of trypsin stopped by adding FCS. Then, cells were recovered and cell cycle analysis is performed as described above.

### Cell viability assays

For 2D cultures, cell proliferation assays were conducted in 96-well culture plates. Assays were run in triplicate. Serial dilutions of compounds were prepared and the viable cell number was determined, on day 4, by the addition of MTS (Promega). Two different experiments were run. Dilutions are as follows: from 1 nM to 200 nM for AZD1152 and paclitaxel and from 100 nM to 1 μM for C5M.

For 3D cultures, the growth was determined by measuring the maximal area of each spheroid and the percentage of expansion at Dx equals (area Dx – area D0/area D0) X 100. IC50 is the concentration that decreases expansion by 2. At the end-point, the number of live cells was determined by the celltiter Glo-3D kit (Promega). IC50 values indicated in Table 1 are the means of both types of determinations, when available. Drug treatments were performed, on day 4 or 6.5 and repeated on 5 spheroids. Three different concentrations were used and, at least, two independent experiments were run.

### Immunofluorescence

Adherent cells grown on glass coverslips were fixed by formaldehyde 4%, for 10 minutes, at 37°C. Immunofluorescence staining was performed as described previously [24]. Immunofluorescence experiments were conducted on whole spheroids as follows. Several spheroids were recovered by pipetting in an eppendorf tube, washed by PBS then fixed by formaldehyde 4%, for several hours, at room temperature or alternatively overnight at 4 °C. Then, spheroids were washed by PBS and permeabilized with PBS, 0.2% Triton-X100, for 30 min. Then, a saturation step with BSA (0.5 mg/ml) preceded the incubation with the antibodies (overnight with mild agitation). Finally, spheroids were washed with TBS-tween 20 (0.5%) and incubated with Cy2-, Cy3- or Hylite 647- fluorescent secondary antibodies for 3 hours, at room temperature. Spheroids were then imaged directly in LabTeck coverslips or fixed with formaldehyde 4% and kept at 4°C.

The antibodies were targeted against the following antigens: α-tubulin (Sigma, 1:1000), phospho-histone H3-Serine10 (Upstate, 1:2000); phospho aurora kinase-A-B-C (Cell signaling technology (CST), 1:100); phospho-AMPK (Thr172; CST, 1:100), phospho-myosin light chain 2 (Ser19; CST 1: 100), mouse P53 (CST 1: 100), Ki-67 (Dako, 1: 100), Cyclin B (Santa cruz, SC-245, 1: 100) and fibronectin (Sigma 1:500). Actin was stained by either phalloidin-rhodamin (1 μg/ml) or phalloidin-Atto 488 or phalloidin-Atto 647. DNA was visualized with either hoechst 33342 (Sigma, 0.5 μg/ml) or NucRed (Thermofischer, one drop in PBS for 15 min at room temperature). Images were collected with a ZEISS 710 Laser Scanning Confocal microscope with either a Plan-Apochromat 20×/0.75 or a water immersion 40 X objectives. Slices of 0.5 μm are shown as well as 3D-projections created by the zen software from Zeiss. A median filter (3/3/1) was applied to most images.

For estimating the percentage of polyploid cells, DNA and polymerized actin were labelled. Then, photos were acquired with a confocal microscope and the scoring was manual. Around 1 000 cells were analysed. For 3D-cultures, the quantification was performed on z-stack images.

### Time lapse

*Ex vivo* experiments were conducted on cells grown on Lab-Tek chambered coverglass (Nalgen Nunc International) and maintained under standard culture conditions (37°C, 5% CO_2_). Spheroids were recovered by pipetting just before imaging and deposited in the Lab-Tek well containing cell culture medium. Images were acquired on a Zeiss dynascope confocal microscope using a Plan-Apochromat 20X / 0.75 objective. Images were analysed with the Zen software provided by Zeiss and a median filter (3/3/1) was applied to all images. At least 10 mitotic cells were simultaneously followed and three independent experiments were conducted.
